# Opportunistic Osteoporosis Screening Reveals Low Bone Density in Patients With Screw Loosening After Lumbar Semi-Rigid Instrumentation: A Case-Control Study

**DOI:** 10.3389/fendo.2020.552719

**Published:** 2021-01-11

**Authors:** Maximilian T. Löffler, Nico Sollmann, Egon Burian, Amirhossein Bayat, Kaywan Aftahy, Thomas Baum, Bernhard Meyer, Yu-Mi Ryang, Jan S. Kirschke

**Affiliations:** ^1^ Department of Diagnostic and Interventional Neuroradiology, School of Medicine, Klinikum rechts der Isar, Technical University of Munich, Munich, Germany; ^2^ Department of Diagnostic and Interventional Radiology, University Medical Center Freiburg, Freiburg im Breisgau, Germany; ^3^ TUM-Neuroimaging Center, Klinikum rechts der Isar, Technical University of Munich, Munich, Germany; ^4^ Department of Diagnostic and Interventional Radiology, University Hospital Ulm, Ulm, Germany; ^5^ Department of Neurosurgery, School of Medicine, Klinikum rechts der Isar, Technical University of Munich, Munich, Germany; ^6^ Department of Neurosurgery, Helios Klinikum Berlin-Buch, Berlin, Germany

**Keywords:** bone mineral density, osteoporosis, spinal fusion and instrumentation, multidetector computed tomography, computer-assisted image analysis, computer neural networks, degenerative spine surgery

## Abstract

**Objective:**

Decreased bone mineral density (BMD) impairs screw purchase in trabecular bone and can cause screw loosening following spinal instrumentation. Existing computed tomography (CT) scans could be used for opportunistic osteoporosis screening for decreased BMD. Purpose of this case-control study was to investigate the association of opportunistically assessed BMD with the outcome after spinal surgery with semi-rigid instrumentation for lumbar degenerative instability.

**Methods:**

We reviewed consecutive patients that had primary surgery with semi-rigid instrumentation in our hospital. Patients that showed screw loosening in follow-up imaging qualified as cases. Patients that did not show screw loosening or—if no follow-up imaging was available (n = 8)—reported benefit from surgery ≥ 6 months after primary surgery qualified as controls. Matching criteria were sex, age, and surgical construct. Opportunistic BMD screening was performed at L1 to L4 in perioperative CT scans by automatic spine segmentation and using asynchronous calibration. Processing steps of this deep learning-driven approach can be reproduced using the freely available online-tool Anduin (https://anduin.bonescreen.de). Area under the curve (AUC) was calculated for BMD as a predictor of screw loosening.

**Results:**

Forty-six elderly patients (69.9 ± 9.1 years)—23 cases and 23 controls—were included. The majority of surgeries involved three spinal motion segments (n = 34). Twenty patients had low bone mass and 13 had osteoporotic BMD. Cases had significantly lower mean BMD (86.5 ± 29.5 mg/cm³) compared to controls (118.2 ± 32.9 mg/cm³, p = 0.001), i.e. patients with screw loosening showed reduced BMD. Screw loosening was best predicted by a BMD < 81.8 mg/cm³ (sensitivity = 91.3%, specificity = 56.5%, AUC = 0.769, p = 0.002).

**Conclusion:**

Prevalence of osteoporosis or low bone mass (BMD ≤ 120 mg/cm³) was relatively high in this group of elderly patients undergoing spinal surgery. Screw loosening was associated with BMD close to the threshold for osteoporosis (< 80 mg/cm³). Opportunistic BMD screening is feasible using the presented approach and can guide the surgeon to take measures to prevent screw loosening and to increase favorable outcomes.

## Introduction

Osteoporosis is the most common metabolic bone disease (1). Predisposing the individual to an increased risk of fracture osteoporosis is characterized by compromised bone strength due to decreased bone mineral density (BMD) (2). With increasing age, a higher incidence of indications for spinal surgery overlaps with a higher prevalence of osteoporosis (3, 4). Therefore, routine osteoporosis screening that can entail fracture prevention and management of osteoporosis is advised in patients aged 50 years and older, especially postmenopausal women (5).

Studies over the last three decades reported failure rates of 13% to 19% for instrumented spine surgery (6–8). Adjacent segment disease (ASD) is a frequent reason for reoperation that is caused by non-physiological stress at the functional segment between a rigid fusion construct and a mobile segment. Semi-rigid instrumentation using the topping-off technique supports the adjacent segments via load sharing by flexible rods while promoting fusion of the lower instrumented segments (9, 10). However, the intended motion in the upper most instrumented segment puts additional forces on the screw-bone interface, which is likely to cause increased rates of screw loosening. Of note, screw loosing is caused by multiple factors that can be categorized into mechanical load and screw-bone purchase. The former is influenced by sagittal balance and body mass index (BMI), the latter by bone quality and BMD.

An association between low BMD and an increased risk of complications and surgical failure rates has been shown in only a few *in-vivo* studies (11, 12), but many *ex-vivo* biomechanical studies investigated this issue (13–19). Surgical failure in patients with decreased bone strength may be due to impaired screw purchase (11), interbody cage subsidence (20), or junctional kyphosis adjacent to the instrumented levels (21, 22). Furthermore, osteoporosis is regarded as a predisposing factor for degenerative spine disease and micro-instability. The prevalence of low bone mass and osteoporosis is relatively high in patients undergoing spinal fusion (4, 23, 24) or spinal surgery in general (3). In conclusion, osteoporosis is believed to be an independent risk factor for instrumentation failure (25), leading to unfavorable outcomes or revision surgery. Awareness of decreased bone strength is important to tackle the challenge of instrumented surgery in the osteoporotic spine.

Preoperative assessment of BMD objectifies doubts about decreased bone strength and can inform the surgical planning process in order to take special measures for osteoporotic conditions. A survey among spine surgeons showed that only 44% of the queried surgeons routinely obtained dual-energy X-ray absorptiometry (DXA) examinations prior to instrumented fusion when osteoporosis was suspected (25). Supplementary DXA examinations may become dispensable if volumetric BMD can be opportunistically assessed in conventional preoperative CT scans. This could help reduce costs and radiation exposure. The feasibility and validity of opportunistic BMD screening in existing CT scans has been extensively shown (26). Here, we present a new screening approach for decreased BMD that involves automatic spine segmentation by a fully convolutional neural network and requires little user interaction (27, 28).

In this case-control study we investigate the association of opportunistically assessed BMD with screw loosening in patients undergoing spinal surgery with semi-rigid instrumentation in the treatment of lumbar degenerative instability. Furthermore, we examine the applicability of a deep-learning driven approach to opportunistic osteoporosis screening in this setting.

## Methods

### Ethics Approval

The present study was approved by the local institutional review board (ethics committee’s reference number 5022/11-A2) and was conducted in accordance with the Declaration of Helsinki. The requirement for informed consent was waived by the institutional review board due to the retrospective character of imaging data collection and post-hoc analysis.

### Patients

To identify eligible patients, we retrospectively searched our hospital information system and picture archiving and communication system (PACS) for patients who underwent primary semi-rigid instrumentation of the lumbosacral spine (i.e. without prior instrumentation) and had clinical follow-up examinations at least 6 months after surgery available. Semi-rigid instrumentation was performed using a pedicle screw–rod system (CD Horizon Legacy, Medtronic) with polyether ether ketone (PEEK) rods using the topping-off approach. The first patients who underwent semi-rigid instrumentation with PEEK rods and this technique were operated on in 2009 at our institution. The total time of retrospective search covered 7 years. Exclusion criteria were 1) no perioperative CT imaging or CT imaging acquired using another than the institutional multidetector CT (MDCT) scanner (29) (because of non-availability of validated HU-to-BMD conversion equations for other devices; n = 6), 2) misplacement of pedicle screws during index surgery (n = 3), and 3) material failure (e.g. screw breakage), ASD, spondylodiscitis, or an incident vertebral fracture in association with screw loosening during follow-up (n = 22). This selection algorithm yielded 97 eligible patients.

To assign each patient to cases or controls, follow-up imaging including CT and radiographs was reviewed for absence/presence of signs of screw loosening by one neuroradiology resident (MTL) using the viewer of the institutional PACS. Based on these readings and clinical follow-up examinations patients were classified as cases or controls. A case showed screw loosening in follow-up imaging. Correspondingly, a control did not show any signs of screw loosening in follow-up imaging or—if there was no follow-up imaging—did report overall benefit from surgery in the latest available clinical examination, taken at the earliest 6 months after index surgery. According to these criteria, out of the 97 patients identified during retrospective search, 52 qualified as cases and 45 as controls.

Eligible cases were matched to controls by sex, age, and surgical construct. Patients who did not offer a matching control were discarded from further analysis. The matching process yielded 23 case patients and 23 controls, forming the final study sample investigated in this study.

### Perioperative Computed Tomography

Preoperative or immediate postoperative CT scans were used for opportunistic BMD screening. All scans were performed on one MDCT scanner (Philips Brilliance 64, Philips Medical Care, Best, The Netherlands). Image data was acquired in helical mode with a peak tube voltage of 120 kVp for standard and 140 kVp for postmyelography studies. Sagittal reformations of the spine with 2 or 3 mm slice thickness were reconstructed using a bone kernel. Daily air calibration was performed to ensure stability of this scanner at -1000 Hounsfield units (HU).

### Automatic Segmentation and Hounsfield Unit Extraction

HU of trabecular bone were extracted in at least one vertebra of L1 to L4. All steps of this semi-automatic procedure were scripted in Python and required little user interaction. Cases, where user interaction was needed, were processed using the freely available online-tool Anduin (https://anduin.bonescreen.de/) (28).

**Figure 1:**
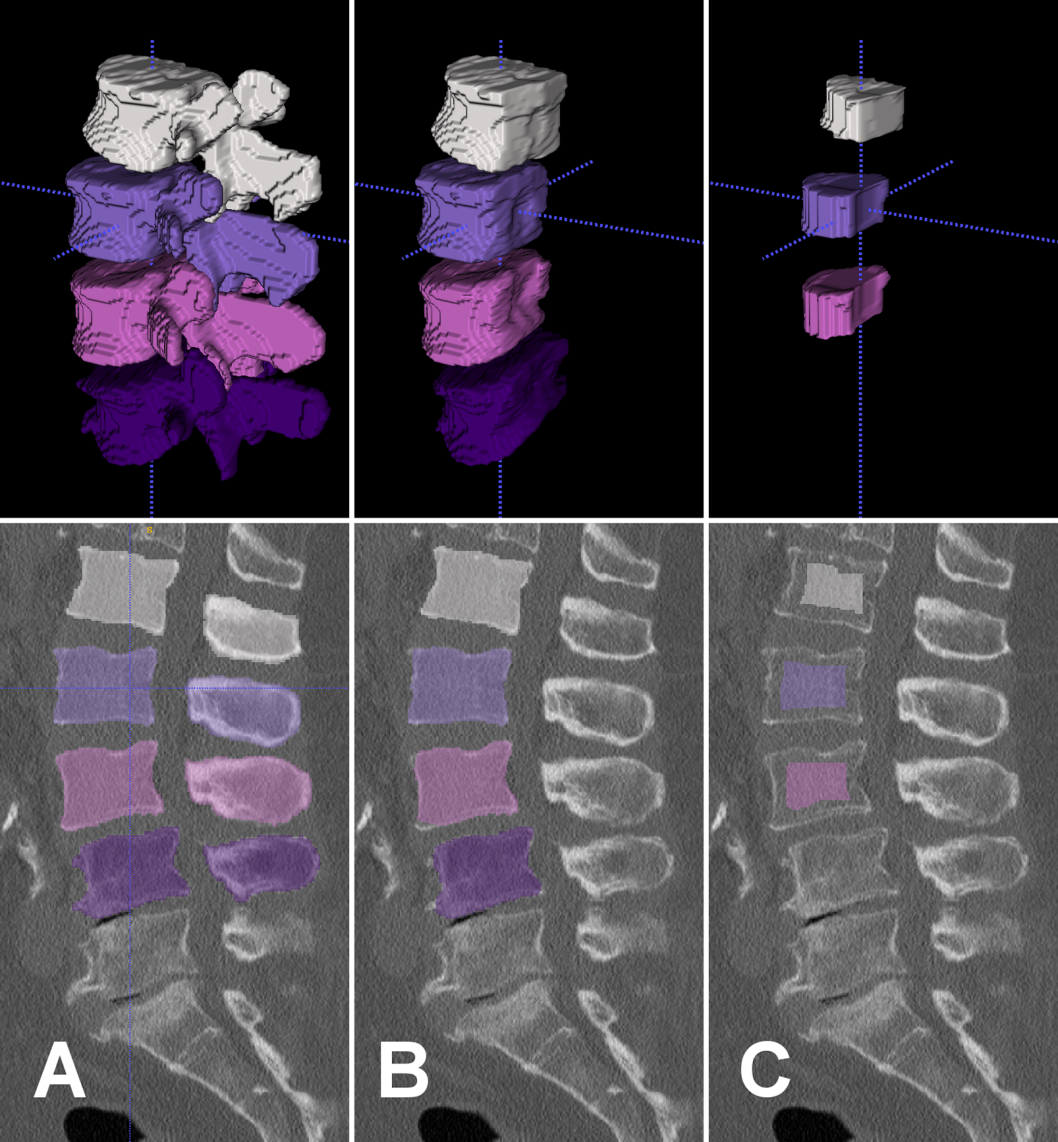
Top row: Segmentation masks in 3D-rendering. Bottom row: Segmentation masks in sagittal view overlaid on preoperative CT scan. Left to right columns: Segmentation masks of L1 to L4 were created after manual labeling of vertebral centroids and automatic segmentation by a fully convolutional network **(A)**. Posterior elements were removed from segmentation masks **(B)**. The outer 10 mm were eroded from segmentation mask and L4 mask was removed due to osteochondrosis **(C)**. These final segmentation masks served as ROIs to extract HU from the CT scan for opportunistic BMD screening. HU, Hounsfield units; BMD, bone mineral density; ROI, region of interest.

**Figure 2:**
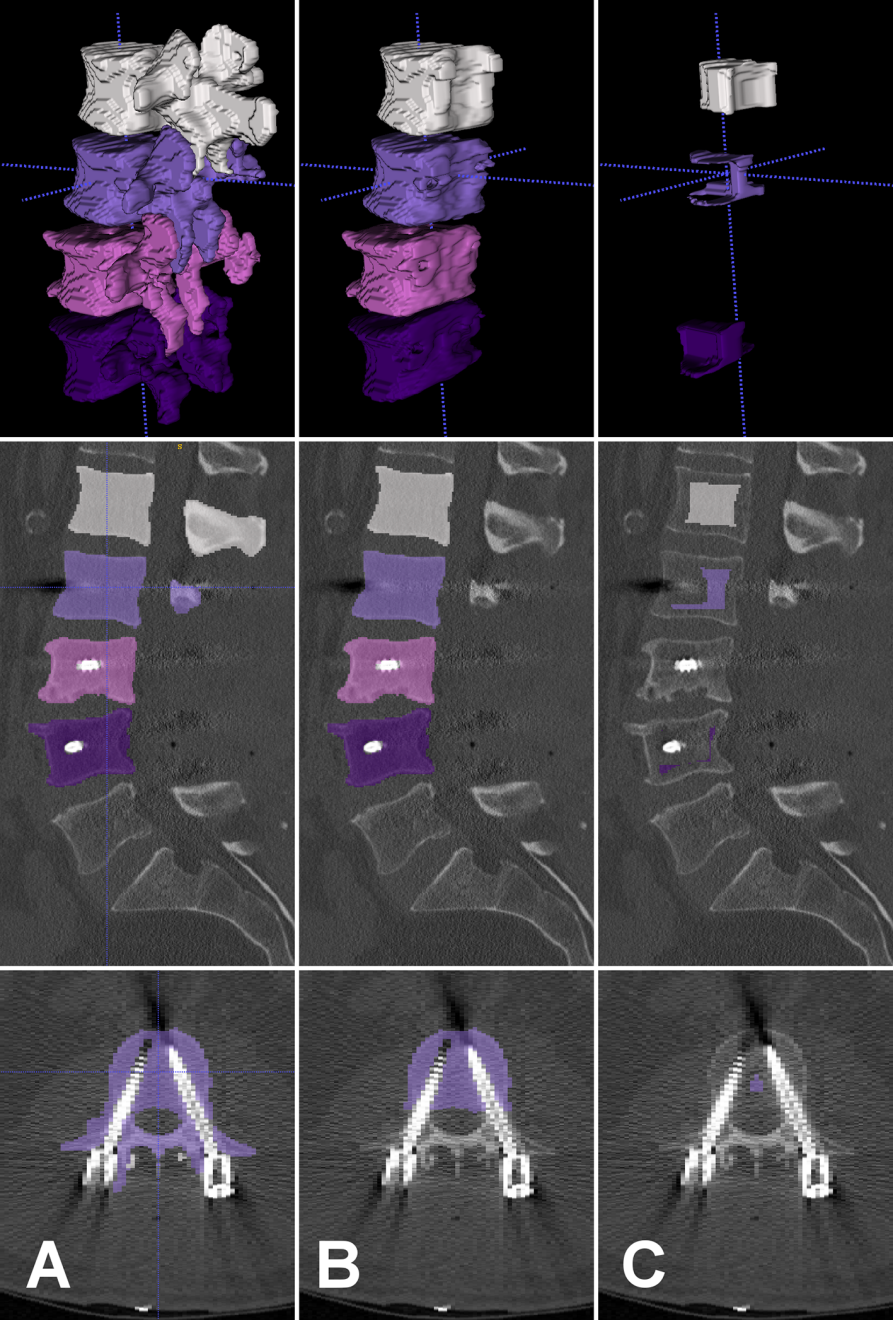
Top to bottom row: Segmentation masks in 3D-rendering, in sagittal view, and in axial view overlaid on postoperative CT scan. Left to right columns: Automatically created segmentation masks of L1 to L4 excluded screw contours by HU thresholding **(A)**. Posterior elements were removed from segmentation masks **(B)**. The outer 10 mm were eroded from segmentation mask and L3 mask was removed due to degenerative changes **(C)**. These final segmentation masks served as ROIs to extract HU from the CT scan for opportunistic BMD screening. HU, Hounsfield units; BMD, bone mineral density; ROI, region of interest.

First, vertebrae were manually labelled and, then, automatically segmented using an in-house developed, fully convolutional neural network (27, 28) (**Figures 1** and **2A**). In case of postoperative scans that depicted vertebrae with pedicle screws the segmentation algorithm excluded any voxels above a threshold of 1400 HU from the segmentation process by default, thus delineating the screw contours and excluding them from vertebral segmentation, which then only covered bone (**Figure 2**). Posterior elements were automatically removed and segmentation masks were eroded by 10 mm to exclude cortical bone or partial volume effects of foreign material (**Figures 1** and **2B, C**). We chose this erosion value after we evaluated several degrees of erosion because it reliably excluded any compact bone that was reaching into the trabecular compartment due to degeneration or small intravertebral herniations.

All CT scans were visually inspected with overlaid eroded segmentation masks using ITK-SNAP software (version 3.8, www.itksnap.org) to identify errors in automatic segmentation and to exclude vertebrae from measurements that were fractured or had severe degenerative alterations (**Figures 1** and **2C**). Mean HU of trabecular bone were extracted from eroded segmentation masks of individual vertebral bodies and averaged over multiple levels if applicable.

### Opportunistic Bone Mineral Density Screening

X-ray attenuation in HU sampled in trabecular bone were converted to volumetric BMD (in mg/cm³) using asynchronous calibration (26). Therefore, HU-to-BMD conversion equations for standard CT studies with 120 kVp and for postmyelography studies with 140 kVp tube voltage were used, which have been previously reported for the used MDCT scanner (29). Following the American College of Radiology (ACR) practice parameters for bone densitometry, osteoporosis was defined as BMD < 80 mg/cm^3^ and low bone mass as 80 mg/cm^3^ ≤ BMD ≤ 120 mg/cm^3^ (30). Throughout this study BMD will refer to volumetric density given in mg/cm³, if not stated otherwise.

### Statistical Analysis

Means of continuous variables were compared with independent samples t-tests assuming equality of variances depending on Levene’s test. Proportions of categorical variables were compared with Pearson’s Chi-squared tests. Area under the curve (AUC) was calculated in receiver operating characteristics (ROC) to test the classification performance of BMD to predict screw loosening. The BMD threshold was determined with maximum Youden’s index. Statistical analyses were conducted with IBM SPSS Statistics 24 (IBM Corp., Armonk, NY, USA). The level of statistical significance was defined as p < 0.05.

## Results

**Figure 3:**
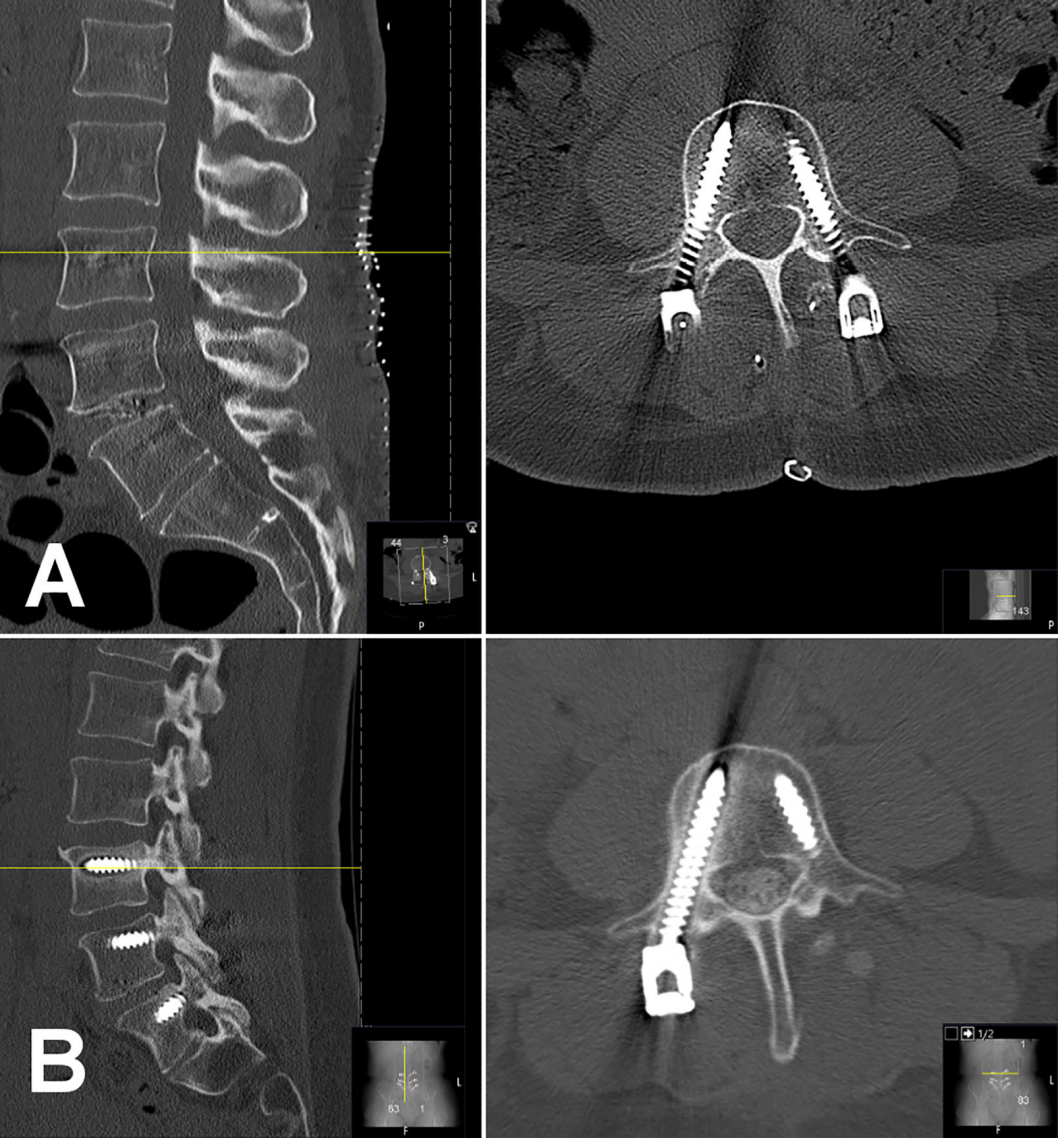
Case of a 48-year-old woman who underwent spinal surgery with semi-rigid instrumentation of levels L4 to S1 for lumbar instability. Of note, this patient has a partially lumbarized S1 vertebra that shows bony fusion at the lateral mass to the os sacrum. Opportunistic BMD evaluation yielded low bone mass (BMD = 95.8 mg/cm³). An immediate postoperative CT scans shows all six pedicle screws at L4, L5, and S1 and an intervertebral cage at L5/S1 in place **(A)**. In 3-months survey the patient reported severe disability according to Oswestry Disability Index (score 42). A control CT scan 178 days after index surgery showed loosening of right L4 screw and both S1 screws (screws not fully depicted; **B**). BMD, bone mineral density.

**Figure 4:**
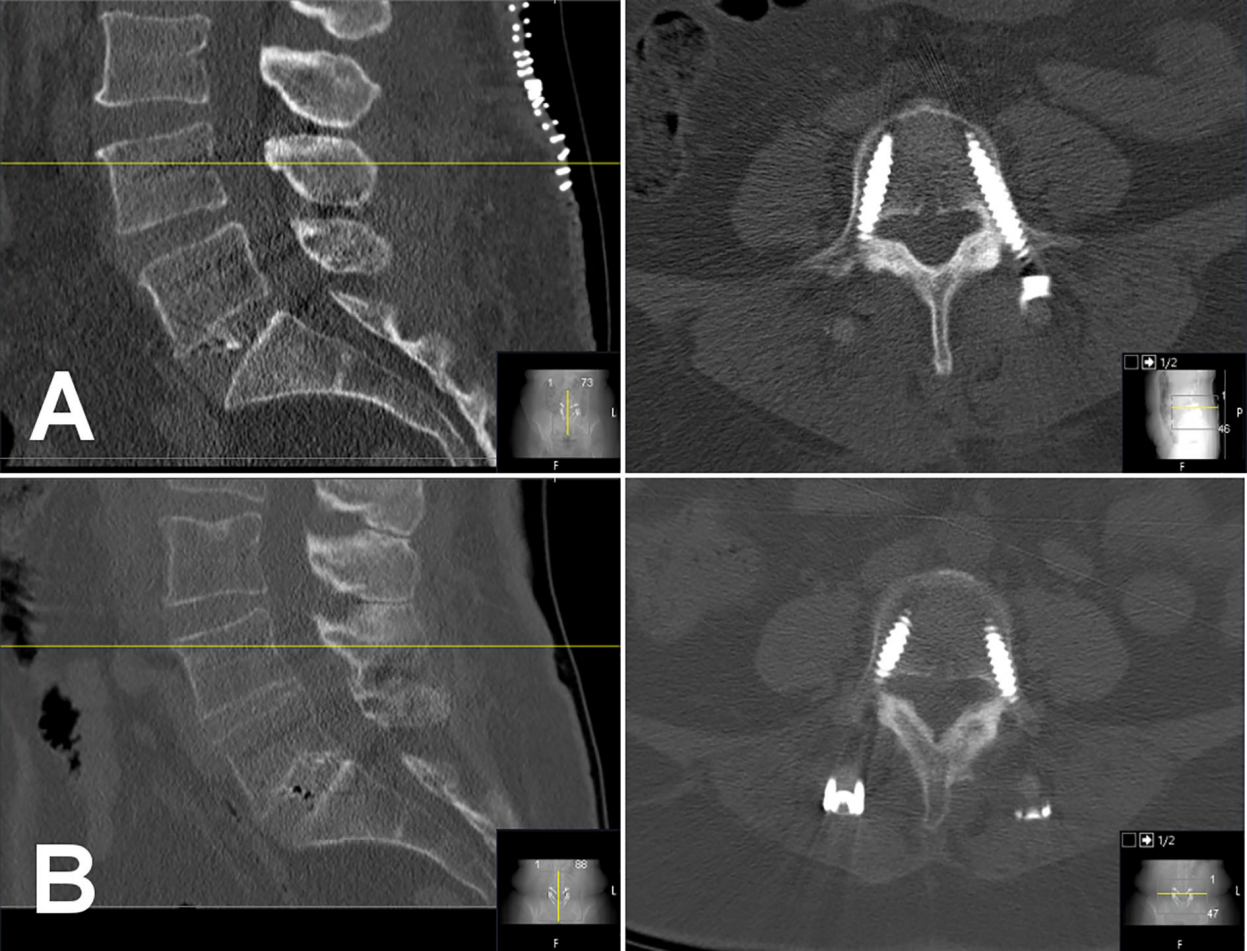
Matched control case of a 48-year-old woman who underwent spinal surgery with semi-rigid instrumentation of levels L4 to S1 for lumbar instability. Opportunistic BMD evaluation yielded normal bone density (BMD = 133.2 mg/cm³). An immediate postoperative CT scans shows all six pedicle screws at L4, L5, and S1 and an intervertebral cage at L5/S1 in place **(A)**. In 12-months survey the patient reported overall benefit from surgery with moderate disability according to Oswestry Disability Index (score 24). A follow-up CT scan more than 7 years after index surgery showed no signs of screw loosening or breakage (screws not fully depicted; **B**). BMD, bone mineral density.

Forty-six elderly patients (mean age = 69.9 ± 9.1 years, range: 48–85 years) that had primary surgery with semi-rigid instrumentation of the lumbar spine were included in this study (**Table 1**). Patients underwent surgery for indications of lumbar instability, spinal stenosis, and spondylolisthesis or any coincidence of these indications. A majority of 34 patients received a fixation construct of 3 motion segments involving L2 to L5 (n = 16) or L3 to S1 (n = 18). Twenty-three patients with radiologically confirmed screw loosening could be matched to 23 controls without signs of screw loosening or who did report overall benefit from surgery at least 6 months after index surgery. The matched groups did not show a significant difference in age, height, weight, BMI, surgical construct, or indication for surgery (p > 0.05). Case patients showed signs of screw loosening after a median follow-up of 185 days (range: 71–1359 days, **Figure 3**). Control patients did not show screw loosening in latest imaging and reported overall benefit from surgery after a median follow-up of 365 days (range: 183–1148 days, **Figure 4**).

**Table 1:** Characteristics of study population stratified by case or control group.

	Cases n = 23	Controls n = 23	Total n = 46
Women, n (%)	14 (61%)	14 (61%)	28 (61%)
Age at operation, years, mean (SD)	68.5 (8.1)	71.4 (10)	69.9 (9.1)
Height, cm, mean (SD)*	171.1 (10.5)	168.7 (8.4)	170 (9.6)
Weight, kg, mean (SD)*	84.4 (15.9)	79.9 (18.7)	82.3 (17.2)
Body mass index, kg/m², mean (SD)*	28.6 (3.3)	27.9 (5.3)	28.3 (4.3)
Indication for surgery, n			
Instability and spinal stenosis	15	17	32
Instability w/o spinal stenosis	6	2	8
Spondylolisthesis with spinal stenosis and instability	1	2	3
Spondylolisthesis w/o spinal stenosis	1	2	3
Surgical construct, n			
L1-5	1	1	2
L2-5	8	8	16
L2-S1	4	4	8
L3-S1	9	9	18
L4-S1	1	1	2
Radiologic follow-up, days, median (range)	185 (71–1359)	229 (8–2679)	191 (8–2679)
Clinical follow-up, days, median (range)	770 (71–2225)	365 (183–1148)	365 (71–2225)

Column means and proportions were not significantly different (p > 0.05); BMD, bone mineral density; *no data available for three control patients.

**Figure 5:**
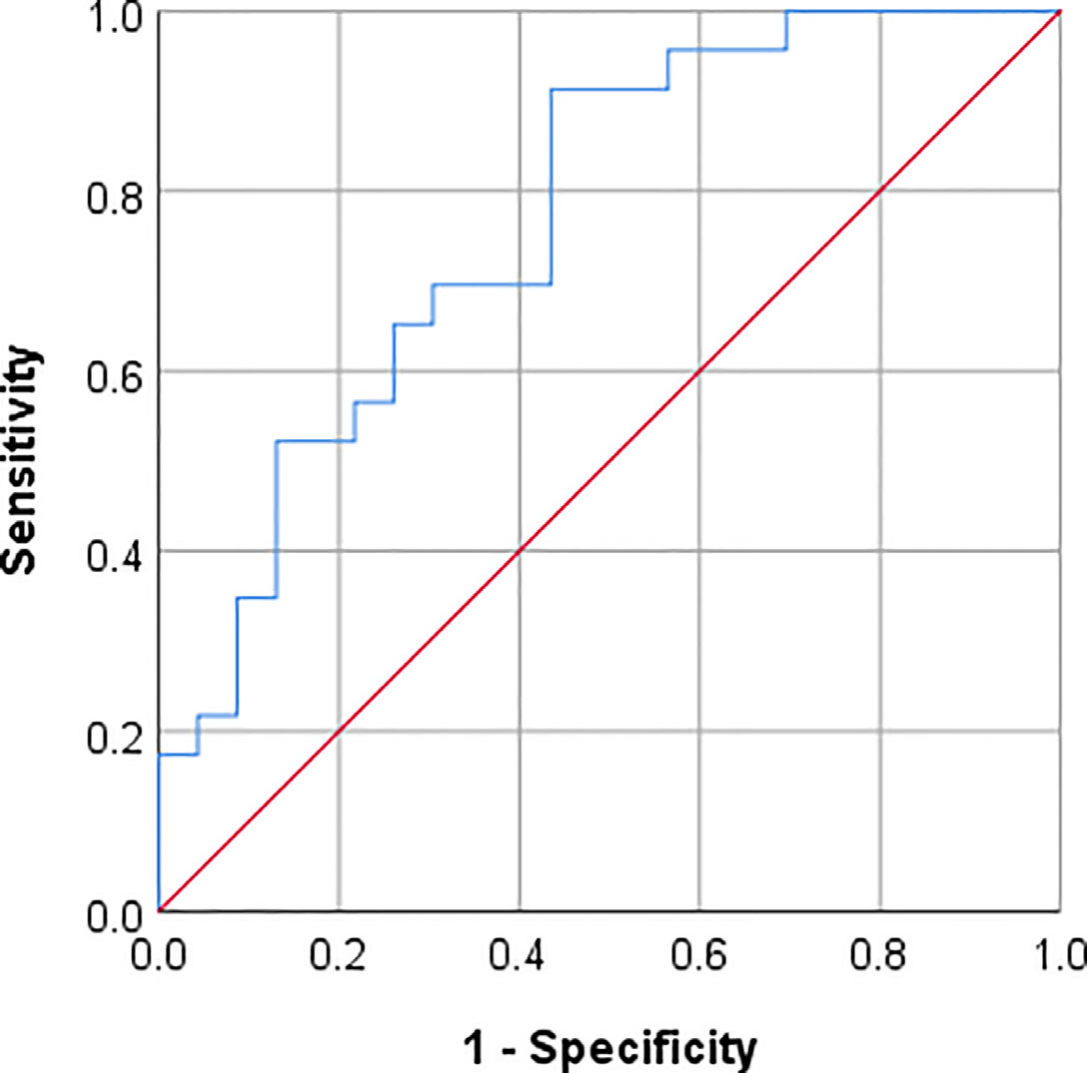
Receiver operating characteristics (ROC) curve for predicting screw loosening after semi-rigid instrumentation by opportunistically assessed BMD at the lumbar spine. BMD was a significant predictor of screw loosening with an AUC = 0.769 (CI: 0.634–0.905, p = 0.002). For BMD < 81.8 mg/cm^3^ screw loosening can be predicted with a sensitivity of 91.3% and specificity of 56.5% (maximum Youden’s index = 0.478). AUC, area under the ROC curve; CI, 95% confidence interval; BMD, bone mineral density.

Opportunistic BMD screening in perioperative CT revealed that case patients had significantly lower mean BMD of 86.5 ± 29.5 mg/cm³ compared to controls with a mean BMD of 118.2 ± 32.9 mg/cm³ (p = 0.001; **Table 2**). Based on the ACR criteria, there were significantly more case patients with osteoporosis (n = 11 cases vs. n = 2 controls, p = 0.003) and significantly fewer with normal BMD (n = 3 cases vs. n = 10 controls, p = 0.022) compared to controls. Moreover, BMD was a significant classifier to predict screw loosening with an AUC of 0.769 (95% confidence interval: 0.634–0.905; p = 0.002; **Figure 5**). The best threshold to predict screw loosening was determined at a BMD < 81.8 mg/cm³ with a sensitivity of 91.3% and specificity of 56.5% (maximum Youden’s index = 0.478). Of note, female patients did not show a significant difference in BMD compared to men (BMD = 98.6 ± 37.0 vs. 108.3 ± 31.3 mg/cm³; p > 0.05).

**Table 2:** BMD characteristics of cases and controls.

	Cases n = 23	Controls n = 23	Cases vs. controls	Total
BMD, mg/cm³, mean (SD)	86.5 (29.5)	118.2 (33)	p = 0.001	102.4 (34.8)
BMD category, n				
Normal	3	10	p = 0.022	13
Low bone mass	9	11	n.s.	20
Osteoporosis	11	2	p = 0.003	13

BMD, bone mineral density; n.s., not statistically significant at p < 0.05.

## Discussion

We conducted a case-control study to investigate whether opportunistically assessed BMD is associated with the risk of screw loosening after spinal surgery with semi-rigid instrumentation for indications of lumbar degeneration. Using a deep-learning driven framework, we showed that opportunistic osteoporosis screening is feasible using perioperative CT scans and allows detection of decreased BMD in patients who were scheduled for spinal surgery. The prevalence of low bone mass or osteoporosis in this elderly group of patients was high with 72%. We were able to demonstrate that patients with screw loosening following spinal surgery had significantly lower BMD by approximately 31.7 mg/cm³ than matched controls without clues to screw loosening who benefited from surgery.

Results of this work are in line with previous studies regarding predisposed short- and long-term surgery-related complications among patients with decreased BMD. A previous study using opportunistic BMD screening showed that consecutive patients in whom follow-up imaging was performed to investigate screw fit after posterior spinal fixation had significantly lower BMD if there were signs of screw loosening compared to patients without screw loosening (29). Furthermore, decreased HU (a surrogate for BMD) in preoperative CT scans has been associated with adjacent vertebral fractures following spinal fusion (12). In 1-year follow-up after posterolateral lumbar fusion, decreased HU in preoperative CT scans were associated with symptomatic pseudarthrosis (31). Moreover, patients with radiographic signs of screw loosening and non-fusion on follow-up imaging after instrumented lumbar fusion had significantly lower areal BMD assessed by DXA compared to patients without these signs (11).

Certainly, screw loosening is a multifactorial process that cannot be reduced to impaired bone strength in osteoporosis. Complex biomechanics of the spine play a significant role and are influenced by load magnitude (BMI) and load direction (sagittal balance) as well as rigid material (32). Of note, BMI showed no significant difference between patients in the case and control groups; information about pre- or postsurgical sagittal balance was not available. Furthermore, low grade infections contribute to screw loosening by a mechanism not entirely understood (33). If the screw-bone interface is compromised due to osteoporotic bone, the surgeon can take measures to increase favorable outcomes after instrumented spinal surgery. For instance, augmented screw fixation using polymethylmethacrylate (PMMA) is recommended in osteoporotic bone (34, 35), because it improves the fixation and fatigue strength according to *ex vivo* investigations (36), reduces the risk of screw loosening and screw pullout (37), and increases fusion rates with maintained correction angles *in vivo* (38). Adapted surgical techniques like PMMA-augmented screw fixations and long-segment constructs are considered to reduce the risk of instrumentation failure in osteoporotic patients (34, 39, 40). Long-segment constructs seem to be beneficial in order to avoid ending within a spinal transition zone or a kyphotic section (22, 41, 42), as these regions are typically prone to ASD, adjacent vertebral body fractures, or implant failure. Moreover, medical treatment with antiresorptive drugs can improve bone strength and, thus, reduce fracture risk (43, 44). Therefore, zoledronic acid infusion reduced the incidence of screw loosening among other surgery-related complications and improved final fusion rates after instrumented lumbar interbody fusion in patients with osteoporosis (45).

The high prevalence of osteoporosis and low bone mass in our study group may be due to the relatively high mean age and relatively more women. Indications for lumbar spine surgery for degenerative disease likely increase in elderly patients and favorable clinical outcomes can be achieved in the most cases regardless of an increased overall surgical risk (46). The relatively high prevalence of decreased BMD is in line with previous studies where approximately 30%–40% of patients above the aged of 50 years undergoing spinal fusion had osteoporotic bone density or fragile bone strength (4, 23). Thus, biomechanical considerations and surgical techniques adapted to the osteoporotic spine become increasingly important when performing spinal instrumentations (40, 42).

The National Osteoporosis Foundation (NOF) recommends all postmenopausal women and men age 50 and older should be evaluated for osteoporosis risk in order to determine the need for BMD testing (5). BMD testing should then be performed using DXA, although opportunistic BMD screening using CT can be equivalent or better to estimate fracture risk (47). Of note, there are many more guidelines for osteoporosis prevention by national and international societies and it is beyond the scope to of this work to discuss them in detail. BMD measurements were not performed on a regular basis prior to surgery in our institution. Here, we performed opportunistic osteoporosis screening in clinical CT scans (48). This method has been investigated in various studies showing good precision (26), which can be further improved if measurements are not performed manually but automatically assisted (49). Therefore, we implemented a deep-learning driven approach to opportunistic osteoporosis screening. Manual interaction was limited to labeling of vertebrae that should be automatically segmented (27, 28) and to the inspection of automatic segmentation masks for quality assurance and exclusion of severely degenerated vertebrae. The latter also reflects a routine necessity for DXA scans—the up-to-date reference standard for bone densitometry (26). Of note, spinal degeneration presents a contra-indication for DXA scans and, if not recognized in the survey scan, measurements can be misinterpreted in the presence of degenerative joint disease (26, 50).

Furthermore, automatic HU measurements had to be converted to BMD using asynchronous calibration (26, 48). This is a necessary step to provide scanner independent BMD values that can be compared to the predefined threshold of low bone mass (80 mg/cm³ ≤ BMD ≤ 120 mg/cm³) and osteoporosis (BMD < 80 mg/cm³) (30). Asynchronous calibration equations for standard 120 kV scans and postmyelography 140 kV scans have been previously established for the MDCT scanner that was also used in this study (29). Good long-term stability can be assumed, even though not previously analyzed for the very same MDCT scanner (47).

We found that patients with screw loosening following spinal surgery had a mean BMD of 86.5 mg/cm³. Moreover, the best diagnostic threshold to predict screw loosening was calculated at a BMD < 81.8 mg/cm³. These values lie close to thresholds reported in biomechanical cadaver studies; therein it was concluded that if BMD is below 80 or 90 mg/cm³, respectively, stability of pedicle screws is insufficient and early screw loosening may be expected, whereas in vertebrae with BMD above 120 mg/cm³ early screw loosening is less likely (16, 18). Furthermore, a previous study hypothesized that an areal BMD below 0.674 ± 0.104 g/cm² indicates a potentially increased risk of spinal fusion failure (11). Although difficult to compare to volumetric BMD, this value certainly lies within the osteoporotic range. Apparently, the mean BMD of cases with screw loosening and the estimated cut-off to predict screw loosening in our study lie within close range to the threshold proposed by the ACR for the diagnosis of osteoporosis (30).

This study has limitations. We report on a relatively small group of 46 matched patients. Their data were collected during clinical routine and according to clinical needs; thus, loss of follow-up due to patients not showing up on scheduled appointments is a possible confounding factor. Whether a patient reports overall benefit from surgery is a subjective criterion that can be dependent on other factors, for example the patient’s expectations. Furthermore, the authors are aware that sagittal balance of the spine is an important biomechanical factor, which can influence the outcome of spinal instrumentation. Unfortunately, long-standing radiographs that would have allowed the analysis of the sagittal vertebral axis before and after surgery were only available for a small part of the presented data since they were not part of the routine perioperative workup in our institution until 2014.

In conclusion, this case-control study showed that decreased BMD may be a highly significant risk factor for screw loosening and unsatisfactory outcomes after spinal surgery with semi-rigid instrumentation for lumbar degenerative instability. Patients with screw loosening in follow-up imaging had decreased BMD close to the diagnostic cut-off for osteoporosis (BMD < 80 mg/cm³). The high prevalence of low bone mass or osteoporosis was likely undetected prior to surgery. Opportunistic BMD screening using the deep-learning driven framework presented here can help to close this diagnostic gap without additional costs. Aware of osteoporotic conditions at the spine the surgeon can use adapted techniques to increase favorable outcomes after semi-rigid instrumentation.

## Data Availability Statement

The datasets presented in this article are not readily available due to ethical considerations in this retrospective study. Requests to access the datasets should be directed to m.loeffler@tum.de.

## Ethics Statement

The studies involving human participants were reviewed and approved by the Ethics Commission of the Technical University of Munich. Written informed consent for participation was not required for this study in accordance with the national legislation and the institutional requirements.

## Author Contributions

ML analyzed and interpreted the patient data. ML, NS, TB, Y-MR, and JK drafted the work or substantively revised it. NS, EB, AB, and KA made substantial contributions to the acquisition of data. NS, BM, Y-MR, and JK made substantial contributions to the conception of the work. All authors contributed to the article and approved the submitted version.

## Funding

This project has received funding from the European Research Council (ERC) under the European Union’s Horizon 2020 research and innovation program (grant agreement No 637164—iBack—ERC-2014-STG) and from the Deutsche Forschungsgemeinschaft (DFG, German Research Foundation; project No 432290010). Furthermore, we acknowledge support by the B. Braun Foundation (project No BBST-D-19-00106).

## Conflict of Interest

The authors declare that the research was conducted in the absence of any commercial or financial relationships that could be construed as a potential conflict of interest.
